# Crystal structure of dicaesium strontium hexa­cyanidoferrate(II), Cs_2_Sr[Fe(CN)_6_], from laboratory X-ray powder data

**DOI:** 10.1107/S2056989020006660

**Published:** 2020-05-22

**Authors:** Nicolas Massoni, Mícheál P. Moloney, Agnès Grandjean, Scott T. Misture, Hans-Conrad zur Loye

**Affiliations:** a CEA, DES, ISEC, DE2D, Univ Montpellier, Marcoule, France; b Kazuo Inamori School of Engineering, Alfred University, Alfred, NY, 14802, USA; c Center for Hierarchical Waste Form Materials, Columbia, SC, 29208, USA

**Keywords:** crystal structure, X-ray powder diffraction, caesium strontium hexa­ferro­cyanate, cryolite-type structure

## Abstract

Ferrocyanides with general formula *A* ^I^ *_x_B* ^II^_*y*_[Fe(CN)_6_], where *A* and *B* are cations, are thought to accept many substitutions on the *A* and *B* positions. In this communication, the synthesis and crystal structure of Cs_2_Sr[Fe(CN)_6_] are reported. The latter was obtained from K_2_Ba[Fe(CN)_6_] particles, put in contact with caesium and strontium ions. Hence, a simultaneous ion-exchange mechanism (Cs for K, Sr for Ba) occurs to yield Cs_2_Sr[Fe(CN)_6_]. The synthesis protocol shows that K_2_BaFe(CN)_6_ particles can be used for the simultaneous trapping of radioactive caesium and strontium nuclides in water streams. Cs_2_Sr[Fe(CN)_6_] adopts the cryolite structure type and is isotypic with the known compound Cs_2_Na[Mn(CN)_6_] [dicaesium sodium hexa­cyanidomanganate(III)]. The octa­hedrally coordinated Sr and Fe sites both are located on inversion centres, and the eightfold-coordinated Cs site on a general position.

## Abstract 2

Cs_2_Sr[Fe(CN)_6_] was synthesized. It is a potential phase to trap simultaneously caesium and strontium radionuclides. The crystal structure is isotypic with Cs_2_Sr[Mn(CN)_6_].

## Chemical context   

Ferrocyanides (FCN), such as Prussian blue, were discovered almost 300 years ago. The attractive properties of these materials for batteries and decontamination processes have ensured that FCNs remain an active research topic (Haas, 1993 ▸; Paolella *et al.*, 2017 ▸). In particular, potassium copper FCN is currently being investigated for the purification of ^137^Cs-contaminated water streams through partial exchange with potassium (Haas, 1993 ▸; Mimura *et al.*, 1997 ▸). To the best of our knowledge, however, using FCNs to extract strontium either alone or with caesium has never been considered before. In the framework of the Center for Hierarchical Waste Forms (CHWM), an Energy Frontier Research Center (EFRC) funded by the US Department of Energy, we have been working on potassium copper FCN as an efficient K-ionic exchanger to capture ^137^Cs and serve as a waste containment matrix (zur Loye *et al.*, 2018 ▸). In this context, we have synthesized a cesium strontium FCN to study its efficiency in immobilizing both ^90^Sr and ^137^Cs, two radionuclides that are in most cases found together in radioactive water streams. Caesium strontium FCNs appear to be poorly described in the ICDD 2020 PDF4+ powder diffraction database (Gates-Rector & Blanton, 2019 ▸). Since the synthesized phase Cs_2_Sr[Fe(CN)_6_] did not match with existing entries, we decided to characterize the structure and we report the results herein.

## Structural commentary   

**Figure 1:**
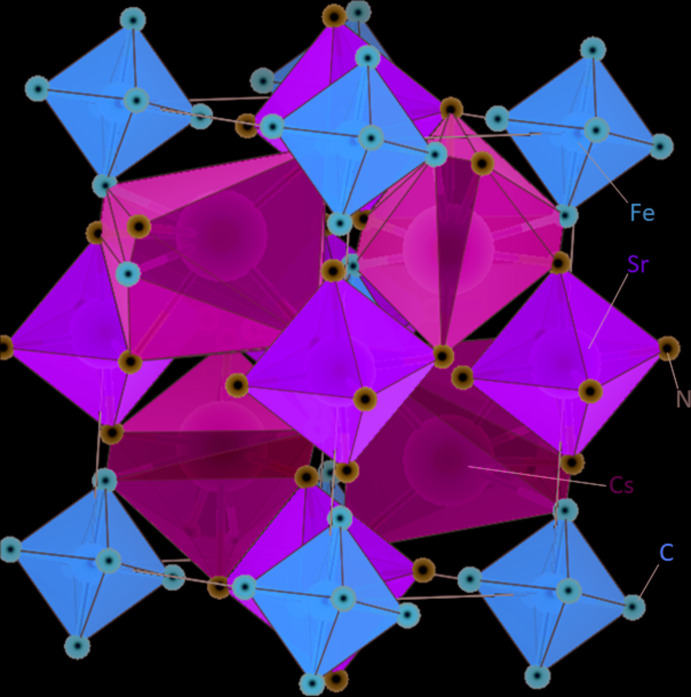
Polyhedral plot of the cryolite-type Cs_2_Sr[Fe(CN)_6_] crystal structure.

Cs_2_Sr[Fe(CN)_6_] is isotypic with Cs_2_Na[Mn(CN)_6_] (Ziegler *et al.*, 1989 ▸). As shown in Table 1 ▸, the lattice parameters of Cs_2_Sr[Fe(CN)_6_] are slightly greater than those of Cs_2_Na[Mn(CN)_6_], but the cell volumes differ by less than 0.3%. The crystal structure adopts the cryolite structure type and comprises a framework of corner-sharing [Sr(CN)_6_] (dark green in Fig. 1 ▸) and [Fe(CN)_6_] octa­hedra (brown in Fig. 1 ▸). Both types of octa­hedra exhibit site symmetry 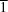, with Sr situated on Wyckoff position 2 *c*, and Fe on 2 *a*. In the voids of this framework, Cs sites (light green in Fig. 1 ▸) have a distorted square-anti­prismatic environment with four C and four N atoms as ligands. The substitution of manganese by iron in Cs_2_Na[Mn(CN)_6_] can be explained by the similar ionic radii of the two elements: *r*_Mn(III)_ = 0.58 Å and *r*_Fe(II)_ = 0.61 Å (Shannon, 1976 ▸). For the substitution of sodium by strontium, the ionic radii differ more substanti­ally: *r*_Na(I)_ = 1.02 Å and *r*_Sr(II)_ = 1.18 Å. The two crystal structures were compared numerically using *COMPSTRU* (de la Flor *et al.*, 2016 ▸). The structure similarity index Δ was calculated to be 0.009 (Bergerhoff *et al.*, 1999 ▸). However, since only a few parameters (11) were refined and many parameters kept fixed in the refinement, the similarity index is not reliable.

## Database survey   

**Figure 2:**
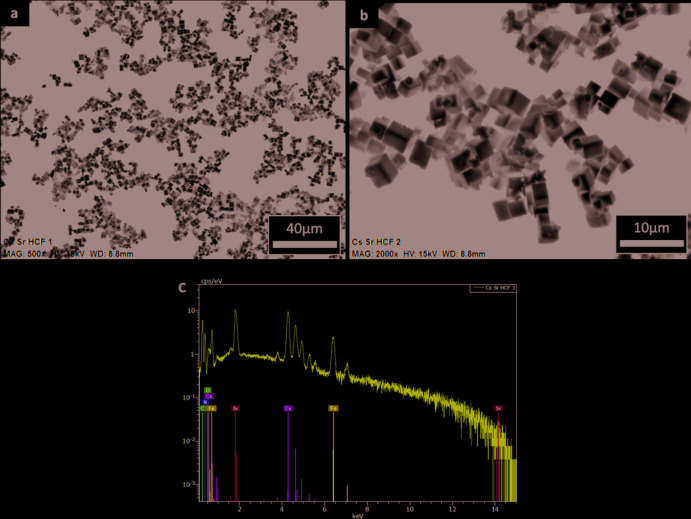
(*a*) and (*b*) SEM-backscattered electron images of Cs_2_Sr[Fe(CN)_6_]. (*c*) EDS spectrum of Cs_2_Sr[Fe(CN)_6_].

Ferrocyanides have rather complex structures. The ICDD 2020 PDF4+ database (Gates-Rector & Blanton, 2019 ▸) contains records of about 1521 phases with the general *A* ^I^ *_x_B* ^II^_*y*_[Fe(CN)_6_] ferrocyanide formula in which *A* and *B* are cations, with no constraints on the *A*:*B* ratio. As shown in Fig. 2 ▸, the studied sample contained only Cs, Sr, Fe, C and N. Hence, we focused on ferrocyanides with *A* ^I^ = Cs and *B* ^II^ = Sr, for which only three phases have been reported, however with poorly described crystal data. The Cs_2_Sr[Fe(CN)_6_] phase studied by Kuznetsov *et al.* (1970 ▸) is reported to crystallize in the tetra­gonal crystal system with *a* ranging from 10.72 to 10.89 Å and *c* from 10.75 to 10.99 Å (PDF00-024-0293 and PDF00-24-0294). The entry for the third phase (PDF00-048-1203), the hydrated ferrocyanide CsSr[Fe(CN)_6_]·3H_2_O reported by Slivko *et al.* (1988 ▸), is comprised only of reflections without further crystal data given. None of these PDF cards matched the X-ray diffraction pattern of the studied sample. As shown in Fig. 2 ▸ *a*,*b*, the cubic crystal habit revealed by SEM measurements hints at a crystal structure with cubic symmetry, but the number of reflections is not consistent with such a highly symmetrical crystal system. The whole pattern can be described by a monoclinic cell and the experimental data are well reproduced by adjusting the reflections from the Cs_2_Na[Mn(CN)_6_] phase (Ziegler *et al.*, 1989 ▸; PDF 04-012-3126). The Cs_2_Sr[Fe(CN)_6_] crystal structure was refined from that of Cs_2_Na[Mn(CN)_6_] assuming complete substitution of manganese by iron and sodium by strontium. As described above, the ionic radii of the corresponding metals are close enough for these substitutions to be possible.

## Synthesis and crystallization   

All solutions were prepared using Millipore water. Cs_2_Sr[Fe(CN)_6_] (Cs_2_SrHCF) particles were not prepared directly by adding Sr and Cs salts to K_4_[Fe(CN)_6_]·3H_2_O. Although it was found that Cs_2_SrHCF could be prepared directly by adding aqueous Sr(NO_3_)_2_ to a K_4_[Fe(CN)_6_]·3H_2_O/CsNO_3_ solution, the yield was extremely poor (≤ 1%). Instead, an ion-exchange reaction was initiated by adding a mixed Sr(NO_3_)_2_/CsNO_3_ solution to K_2_Ba[Fe(CN)_6_] particles, thereby simultaneously substituting barium for strontium and potassium for cesium. This simple approach, using K_2_Ba[Fe(CN)_6_]·2.6H_2_O (K_2_BaHCF) as an inter­mediate compound, allowed 1:1 amounts of Cs_2_SrHCF to be produced from K_2_BaHCF by ion exchange.

**Figure 3:**
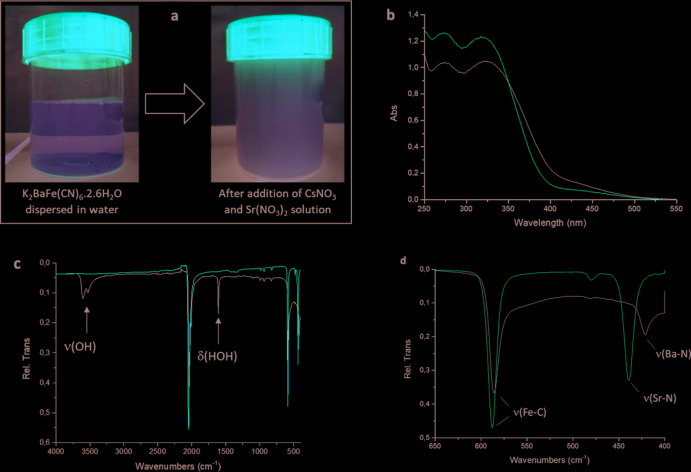
(*a*) Pictures of a clear yellow K_2_Ba[Fe(CN)_6_]·2.6H_2_O dispersion before (left) and immediately after (right) the addition of Cs/Sr. (*b*) UV–Vis spectra of K_2_Ba[Fe(CN)_6_]·2.6H_2_O (black) and Cs_2_Sr[Fe(CN)_6_] (red). Both spectra show the characteristic features of the [Fe(CN)_6_] moiety (Gray & Beach, 1963 ▸). (*c*) FT–IR spectra of K_2_BaHCF (black) and Cs_2_SrHCF (red). All peaks shift to higher frequencies after ion exchange. The arrows indicate the *δ*(HOH) signal at 1611 cm^−1^ and *ν*(OH) signals at 3527 cm^−1^ and 3601 cm^−1^ observed for K_2_BaHCF but not for Cs_2_SrHCF. These signals indicate the presence of structural water, which is common in hexa­cyanidoferrate particles. (*d*) Enlarged view of the FT–IR spectra in the 400–650 cm^−1^ range.

Briefly, the K_2_BaHCF itself was prepared by adding a 1.5 *M* solution of Ba(NO_3_)_2_ to a 1 *M* solution of K_4_[Fe(CN)_6_]·3H_2_O as described by Padigi *et al.* (2015 ▸). Once prepared, K_2_BaHCF was collected by centrifugation, washed and dried. Its chemical composition (K, Fe and Ba) and water content, respectively, were determined by inductively coupled plasma (ICP) analysis and thermogravimetric analysis (TGA). The dried K_2_BaHCF particles redispersed readily in water, producing a clear, slightly yellow dispersion. Cs_2_SrHCF forms immediately as a milky white precipitate (Fig. 3 ▸ *a*) upon adding the mixed CsNO_3_/Sr(NO_3_)_2_ solution to the clear yellow K_2_BaHCF dispersion. To ensure complete substitution, 2.2 moles of CsNO_3_ and 1.1 moles of Sr(NO_3_)_2_ were added for every mole of K_2_BaHCF present. After being left to mix for 1 h, the formed Cs_2_SrHCF was collected by centrifugation, washed and dried. The chemical composition (Fe and Sr) of the powder was determined by ICP analysis while the Cs content was determined by atomic absorption spectroscopy (AAS). An initial characterization of the Cs_2_SrHCF powder was carried out by TGA, UV–Vis and FT–IR spectroscopy. The UV–Vis spectrum of the Cs_2_SrHCF (Fig. 3 ▸ *b*) confirmed that the [Fe(CN)_6_] moiety was maintained with only slight decreases in the wavelengths of the various absorption peaks (Gray & Beach, 1963 ▸). The FT–IR spectra of Cs_2_SrHCF and K_2_BaHCF are shown in Fig. 3 ▸ *c*. While a *δ*(HOH) signal is observed for K_2_BaHCF at 1611 cm^−1^ along with *ν*(OH) signals at 3527 cm^−1^ and 3601 cm^−1^, no such signals were detected for Cs_2_SrHCF. This absence of water was confirmed by TGA, which showed no mass loss between 30 and 400°C (Fig. S1 in the supporting information. The largest change was in the *ν*(*M*—N) stretching mode, which shifted from 421 cm^−1^ ν(Ba—N) to 439 cm^−1^ *ν*(Sr—N) (Fig. 3 ▸ *d*).

## Refinement   

**Figure 4:**
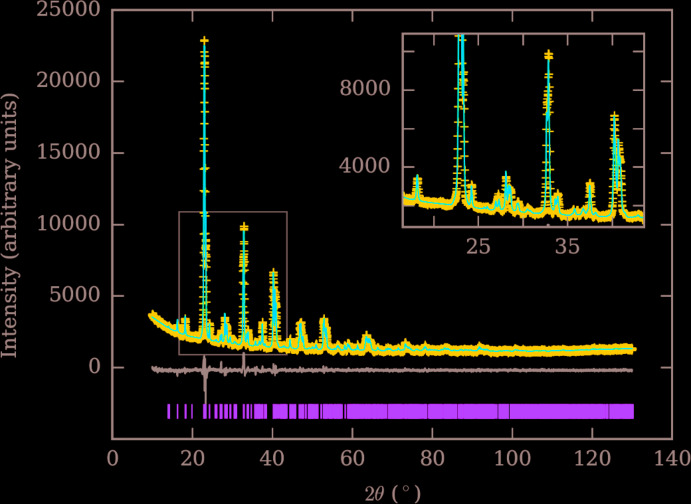
Observed and calculated X-ray powder diffraction intensities for Cs_2_Sr[Fe(CN)_6_].

Crystal data and details of the data collection and structure refinement methods are summarized in Table 2 ▸. The observed and calculated intensities are shown in Fig. 4 ▸ along with the difference pattern. For the refinement of Cs_2_Sr[Fe(CN)_6_], atomic positions of the Cs_2_Na[Mn(CN)_6_] phase (Ziegler *et al.*, 1989 ▸) and the given individual isotropic displacement parameters were used. All occupancies were set to unity because of the experimentally determined composition. Except for cesium, all displacement parameters were kept fixed because otherwise some became negative. The positions of the nitro­gen and carbon atoms were also kept fixed. Since iron and strontium atoms are in special positions, only the lattice parameters, the position of the cesium atom and its *U*_iso_ value were refined, together with three profile parameters. The residual electron density is about is 6.06 e Å^−3^ at a distance of 0.71 Å from Cs.

## Supplementary Material

Crystal structure: contains datablock(s) cacesio, I. DOI: 10.1107/S2056989020006660/wm5555sup1.cif

TG curves of Cs2Sr[Fe(CN)6] and K2Ba[Fe(CN)6].2.6H2O. DOI: 10.1107/S2056989020006660/wm5555sup2.png

CCDC reference: 2004551

Additional supporting information:  crystallographic information; 3D view; checkCIF report

## Figures and Tables

**Table 1:** Comparison of lattice parameters (Å, °), selected bond lengths (Å) and volumes (Å^3^) for Cs_2_Sr[Fe(CN)_6_] and Cs_2_Na[Mn(CN)_6_]

	Cs_2_Sr[Fe(CN)_6_]		Cs_2_Na[Mn(CN)_6_]
*a*, *b*, *c*	7.6237 (2), 7.7885 (2), 10.9600 (3)	*a*, *b*, *c*	7.597 (1), 7.806 (1), 10.950 (1)
α, β, γ	90, 90.4165 (19), 90	α, β, γ	90, 90.07 (1), 90
*V*	650.76 (4)	*V*	649.36
			
Cs polyhedron volume*	44.56	Cs polyhedron volume*	44.46
Cs—C1	3.603 (4)	Cs—C1	3.6291 (5)
Cs—N1	3.348 (4)	Cs—N1	3.3688 (5)
Cs—C1^i^	3.628 (3)	Cs—C1^i^	3.5936 (5)
Cs—N1^i^	3.5232 (18)	Cs—N1^i^	3.4920 (5)
Cs—C2^ii^	3.592 (6)	Cs—C2^ii^	3.5831 (4)
Cs—N2^ii^	3.367 (6)	Cs—N2^ii^	3.3839 (3)
Cs—C3^iii^	3.711 (5)	Cs—C3^iii^	3.7044 (4)
Cs—N3^iii^	3.274 (6)	Cs—N3^iii^	3.2840 (3)
Sr octa­hedron volume*	20.49	Na octa­hedron volume*	20.45
Fe octa­hedron volume*	10.49	Mn octa­hedron volume*	10.47

*Atomic bond lengths were not compared since the positions of C, N, Fe and Sr were not refined. Volumes were calculated by *VESTA* V3.2.1 (Momma & Izumi, 2011 ▸). [Symmetry codes: (i) −*x* + 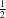, *y* − 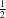, −*z* + 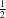; (ii) *x* + 1, *y*, *z*; (iii) *x* + 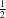, −*y* + 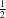, *z* + 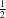.]

**Table 2:** Experimental details

Crystal data	
Chemical formula	Cs_2_Sr[Fe(CN)_6_]
*M*_r_	565.4
Crystal system, space group	Monoclinic, *P*2_1_/*n*
Temperature (K)	293
*a*, *b*, *c* (Å)	7.6237 (2), 7.7885 (2), 10.9600 (3)
β (°)	90.4165 (19)
*V* (Å^3^)	650.76 (4)
*Z*	2
Radiation type	Cu *K*α_1_, λ = 1.540562, 1.544390 Å
Specimen shape, size (mm)	Flat sheet, 25 × 25

Data collection	
Diffractometer	Panalytical XPert MPD Pro
Specimen mounting	Packed powder pellet
Data collection mode	Reflection
Scan method	Step
2θ values (°)	2θ_min_ = 10.023 2θ_max_ = 130.010 2θ_step_ = 0.017

Refinement	
*R* factors and goodness of fit	*R*_p_ = 0.031, *R*_wp_ = 0.043, *R*_exp_ = 0.025, *R*(*F*) = 0.101, χ^2^ = 2.993
No. of parameters	11

Coordinates from an isotypic compound. Computer programs: *Data Collector* (Panalytical, 2011 ▸), *JANA2006* (Petříček *et al.*, 2014 ▸), *VESTA* (Momma & Izumi, 2011 ▸) and *publCIF* (Westrip, 2010 ▸).

## supplementary crystallographic information

### Crystal data

**Table:**

Cs_2_Sr[Fe(CN)_6_]	*F*(000) = 504
*M**_r_* = 565.4	y
Monoclinic, *P*2_1_/*n*	*D*_x_ = 2.885 Mg m^−^^3^
*a* = 7.6237 (2) Å	Cu *K*α_1_ radiation, λ = 1.540562, 1.544390 Å
*b* = 7.7885 (2) Å	*T* = 293 K
*c* = 10.9600 (3) Å	Particle morphology: plate-like
β = 90.4165 (19)°	brown
*V* = 650.76 (4) Å^3^	flat_sheet, 25 × 25 mm
*Z* = 2	Specimen preparation: Prepared at 1873 K and 100 kPa, cooled at 30 K min^−^^1^

### Data collection

**Table:**

Panalytical XPert MPD Pro diffractometer	Data collection mode: reflection
Radiation source: sealed X-ray tube	Scan method: step
Specimen mounting: packed powder pellet	2θ_min_ = 10.023°, 2θ_max_ = 130.010°, 2θ_step_ = 0.017°

### Refinement

**Table:**

*R*_p_ = 0.031	11 parameters
*R*_wp_ = 0.043	0 restraints
*R*_exp_ = 0.025	26 constraints
*R*(*F*) = 0.101	Weighting scheme based on measured s.u.'s
6881 data points	(Δ/σ)_max_ = 0.012
Profile function: Lorentzian	Background function: Manual background

### Special details

**Table:**

Refinement. The Platon test reports 21 Alerts level C. They could be gathered in three groups for explanation :i)17 C-Alerts (out of 21) are "missing esd on x,y,z coordinates of N and C atoms. This is normal since these positions were not refined. Hence no esd was calculated by Jana2006.ii)3 C-Alerts (out of 21) are due to a slighlty larger Fourier difference density than allowed by CheckCIF. I can not enhance the quality of the data so no reduction of this value can be done.iii) The last C-Alert is about a "calc. and reported Sum Formula which differ. I did not find the origin of the Alert.

### Fractional atomic coordinates and isotropic or equivalent isotropic displacement parameters (Å^2^)

**Table:**

	*x*	*y*	*z*	*U*_iso_*/*U*_eq_	
Cs	0.5080 (4)	−0.04022 (18)	0.2497 (7)	0.0410 (6)*	
Sr	0	0	0.5	0.024*	
Fe	0	0	0	0.019*	
C1	0.0488	0.0087	0.178	0.029*	
N1	0.0754	0.0169	0.2809	0.052*	
C2	−0.2128	0.1444	0.022	0.03*	
N2	−0.3347	0.2296	0.0368	0.048*	
C3	0.1449	0.2097	−0.0251	0.029*	
N3	0.2281	0.3301	−0.0406	0.046*	

### Atomic displacement parameters (Å^2^)

**Table:**

	*U*^11^	*U*^22^	*U*^33^	*U*^12^	*U*^13^	*U*^23^
Cs	0.0576 (17)	0.0343 (13)	0.0345 (16)	−0.016 (4)	0.0050 (17)	−0.008 (4)

### Geometric parameters (Å, º)

**Table:**

Cs—C1	3.603 (3)	Sr—N3^i^	2.4965 (1)
Cs—C1^i^	3.628 (2)	Sr—N3^vi^	2.4965 (1)
Cs—N1	3.348 (3)	Fe—C1	1.9845 (1)
Cs—N1^i^	3.5232 (18)	Fe—C1^vii^	1.9845 (1)
Cs—C2^ii^	3.592 (6)	Fe—C2	1.9901 (1)
Cs—N2^ii^	3.367 (6)	Fe—C2^vii^	1.9901 (1)
Cs—C3^iii^	3.711 (5)	Fe—C3	1.9920 (1)
Cs—N3^iii^	3.274 (6)	Fe—C3^vii^	1.9920 (1)
Sr—N1	2.4767 (1)	C1—N1	1.1462 (1)
Sr—N1^iv^	2.4767 (1)	C2—N2	1.1543 (1)
Sr—N2^v^	2.4857 (1)	C3—N3	1.1455 (1)
Sr—N2^iii^	2.4857 (1)		
			
Cs^i^—Cs—Cs^viii^	89.42 (6)	N1—Cs—N3^iii^	111.23 (16)
Cs^i^—Cs—Cs^ix^	88.75 (4)	N1^i^—Cs—C2^ii^	115.67 (16)
Cs^i^—Cs—Cs^x^	178.16 (6)	N1^i^—Cs—N2^ii^	127.6 (2)
Cs^i^—Cs—Cs^xi^	94.04 (12)	N1^i^—Cs—C3^iii^	142.4 (2)
Cs^i^—Cs—Cs^xii^	93.31 (12)	N1^i^—Cs—N3^iii^	130.34 (17)
Cs^i^—Cs—Sr	57.71 (7)	C2^ii^—Cs—N2^ii^	18.74 (3)
Cs^i^—Cs—Sr^ii^	128.17 (12)	C2^ii^—Cs—C3^iii^	91.10 (5)
Cs^i^—Cs—Sr^i^	55.62 (7)	C2^ii^—Cs—N3^iii^	89.11 (6)
Cs^i^—Cs—Sr^viii^	125.77 (11)	N2^ii^—Cs—C3^iii^	85.88 (4)
Cs^i^—Cs—C1	52.06 (4)	N2^ii^—Cs—N3^iii^	89.50 (5)
Cs^i^—Cs—C1^i^	39.87 (4)	C3^iii^—Cs—N3^iii^	17.47 (3)
Cs^i^—Cs—N1	52.61 (4)	Cs^xiii^—Sr—Cs	108.15 (11)
Cs^i^—Cs—N1^i^	35.27 (4)	Cs^xiii^—Sr—Cs^i^	67.33 (6)
Cs^i^—Cs—C2^ii^	135.00 (18)	Cs^xiii^—Sr—Cs^viii^	71.79 (7)
Cs^i^—Cs—N2^ii^	134.15 (18)	Cs^xiii^—Sr—Cs^iv^	71.85 (11)
Cs^i^—Cs—C3^iii^	133.13 (17)	Cs^xiii^—Sr—Cs^xii^	180
Cs^i^—Cs—N3^iii^	135.35 (19)	Cs^xiii^—Sr—Cs^xiv^	108.21 (7)
Cs^viii^—Cs—Cs^ix^	178.16 (6)	Cs^xiii^—Sr—Cs^vi^	112.67 (6)
Cs^viii^—Cs—Cs^x^	88.75 (4)	Cs^xiii^—Sr—N1	67.84 (8)
Cs^viii^—Cs—Cs^xi^	84.87 (11)	Cs^xiii^—Sr—N1^iv^	112.16 (8)
Cs^viii^—Cs—Cs^xii^	84.16 (11)	Cs^xiii^—Sr—N2^v^	55.97 (3)
Cs^viii^—Cs—Sr	51.21 (7)	Cs^xiii^—Sr—N2^iii^	124.03 (3)
Cs^viii^—Cs—Sr^ii^	121.54 (11)	Cs^xiii^—Sr—N3^i^	137.39 (4)
Cs^viii^—Cs—Sr^i^	123.59 (12)	Cs^xiii^—Sr—N3^vi^	42.61 (4)
Cs^viii^—Cs—Sr^viii^	53.50 (6)	Cs—Sr—Cs^i^	68.79 (6)
Cs^viii^—Cs—C1	40.22 (4)	Cs—Sr—Cs^viii^	73.18 (7)
Cs^viii^—Cs—C1^i^	126.52 (9)	Cs—Sr—Cs^iv^	180
Cs^viii^—Cs—N1	37.42 (4)	Cs—Sr—Cs^xii^	71.85 (11)
Cs^viii^—Cs—N1^i^	124.14 (8)	Cs—Sr—Cs^xiv^	106.82 (7)
Cs^viii^—Cs—C2^ii^	98.28 (9)	Cs—Sr—Cs^vi^	111.21 (6)
Cs^viii^—Cs—N2^ii^	79.54 (8)	Cs—Sr—N1	41.51 (8)
Cs^viii^—Cs—C3^iii^	73.00 (7)	Cs—Sr—N1^iv^	138.49 (8)
Cs^viii^—Cs—N3^iii^	90.47 (9)	Cs—Sr—N2^v^	105.29 (4)
Cs^ix^—Cs—Cs^x^	93.09 (6)	Cs—Sr—N2^iii^	74.71 (4)
Cs^ix^—Cs—Cs^xi^	95.39 (13)	Cs—Sr—N3^i^	52.52 (6)
Cs^ix^—Cs—Cs^xii^	95.81 (13)	Cs—Sr—N3^vi^	127.48 (6)
Cs^ix^—Cs—Sr	127.44 (12)	Cs^i^—Sr—Cs^viii^	109.57 (11)
Cs^ix^—Cs—Sr^ii^	59.69 (7)	Cs^i^—Sr—Cs^iv^	111.21 (6)
Cs^ix^—Cs—Sr^i^	55.27 (7)	Cs^i^—Sr—Cs^xii^	112.67 (6)
Cs^ix^—Cs—Sr^viii^	127.94 (12)	Cs^i^—Sr—Cs^xiv^	70.43 (11)
Cs^ix^—Cs—C1	138.10 (8)	Cs^i^—Sr—Cs^vi^	180
Cs^ix^—Cs—C1^i^	51.68 (4)	Cs^i^—Sr—N1	61.18 (7)
Cs^ix^—Cs—N1	140.75 (6)	Cs^i^—Sr—N1^iv^	118.82 (7)
Cs^ix^—Cs—N1^i^	54.06 (4)	Cs^i^—Sr—N2^v^	36.60 (5)
Cs^ix^—Cs—C2^ii^	83.13 (9)	Cs^i^—Sr—N2^iii^	143.40 (5)
Cs^ix^—Cs—N2^ii^	101.85 (11)	Cs^i^—Sr—N3^i^	70.10 (5)
Cs^ix^—Cs—C3^iii^	108.22 (12)	Cs^i^—Sr—N3^vi^	109.90 (5)
Cs^ix^—Cs—N3^iii^	90.74 (10)	Cs^viii^—Sr—Cs^iv^	106.82 (7)
Cs^x^—Cs—Cs^xi^	85.93 (12)	Cs^viii^—Sr—Cs^xii^	108.21 (7)
Cs^x^—Cs—Cs^xii^	86.37 (12)	Cs^viii^—Sr—Cs^xiv^	180
Cs^x^—Cs—Sr	120.84 (11)	Cs^viii^—Sr—Cs^vi^	70.43 (11)
Cs^x^—Cs—Sr^ii^	52.95 (7)	Cs^viii^—Sr—N1	51.05 (8)
Cs^x^—Cs—Sr^i^	125.72 (13)	Cs^viii^—Sr—N1^iv^	128.95 (8)
Cs^x^—Cs—Sr^viii^	52.97 (6)	Cs^viii^—Sr—N2^v^	124.75 (7)
Cs^x^—Cs—C1	126.21 (7)	Cs^viii^—Sr—N2^iii^	55.25 (7)
Cs^x^—Cs—C1^i^	141.80 (10)	Cs^viii^—Sr—N3^i^	122.47 (4)
Cs^x^—Cs—N1	125.56 (5)	Cs^viii^—Sr—N3^vi^	57.53 (4)
Cs^x^—Cs—N1^i^	146.56 (9)	Cs^iv^—Sr—Cs^xii^	108.15 (11)
Cs^x^—Cs—C2^ii^	45.51 (8)	Cs^iv^—Sr—Cs^xiv^	73.18 (7)
Cs^x^—Cs—N2^ii^	45.53 (9)	Cs^iv^—Sr—Cs^vi^	68.79 (6)
Cs^x^—Cs—C3^iii^	46.01 (8)	Cs^iv^—Sr—N1	138.49 (8)
Cs^x^—Cs—N3^iii^	44.55 (9)	Cs^iv^—Sr—N1^iv^	41.51 (8)
Cs^xi^—Cs—Cs^xii^	166.72 (4)	Cs^iv^—Sr—N2^v^	74.71 (4)
Cs^xi^—Cs—Sr	123.46 (9)	Cs^iv^—Sr—N2^iii^	105.29 (4)
Cs^xi^—Cs—Sr^ii^	126.26 (10)	Cs^iv^—Sr—N3^i^	127.48 (6)
Cs^xi^—Cs—Sr^i^	59.14 (9)	Cs^iv^—Sr—N3^vi^	52.52 (6)
Cs^xi^—Cs—Sr^viii^	50.43 (7)	Cs^xii^—Sr—Cs^xiv^	71.79 (7)
Cs^xi^—Cs—C1	75.93 (12)	Cs^xii^—Sr—Cs^vi^	67.33 (6)
Cs^xi^—Cs—C1^i^	108.98 (13)	Cs^xii^—Sr—N1	112.16 (8)
Cs^xi^—Cs—N1	94.11 (14)	Cs^xii^—Sr—N1^iv^	67.84 (8)
Cs^xi^—Cs—N1^i^	90.80 (13)	Cs^xii^—Sr—N2^v^	124.03 (3)
Cs^xi^—Cs—C2^ii^	43.39 (9)	Cs^xii^—Sr—N2^iii^	55.97 (3)
Cs^xi^—Cs—N2^ii^	41.07 (9)	Cs^xii^—Sr—N3^i^	42.61 (4)
Cs^xi^—Cs—C3^iii^	125.88 (9)	Cs^xii^—Sr—N3^vi^	137.39 (4)
Cs^xi^—Cs—N3^iii^	130.40 (11)	Cs^xiv^—Sr—Cs^vi^	109.57 (11)
Cs^xii^—Cs—Sr	52.97 (8)	Cs^xiv^—Sr—N1	128.95 (8)
Cs^xii^—Cs—Sr^ii^	55.18 (8)	Cs^xiv^—Sr—N1^iv^	51.05 (8)
Cs^xii^—Cs—Sr^i^	133.85 (9)	Cs^xiv^—Sr—N2^v^	55.25 (7)
Cs^xii^—Cs—Sr^viii^	116.50 (7)	Cs^xiv^—Sr—N2^iii^	124.75 (7)
Cs^xii^—Cs—C1	100.15 (13)	Cs^xiv^—Sr—N3^i^	57.53 (4)
Cs^xii^—Cs—C1^i^	83.65 (13)	Cs^xiv^—Sr—N3^vi^	122.47 (4)
Cs^xii^—Cs—N1	81.64 (13)	Cs^vi^—Sr—N1	118.82 (7)
Cs^xii^—Cs—N1^i^	101.55 (14)	Cs^vi^—Sr—N1^iv^	61.18 (7)
Cs^xii^—Cs—C2^ii^	131.46 (10)	Cs^vi^—Sr—N2^v^	143.40 (5)
Cs^xii^—Cs—N2^ii^	128.90 (10)	Cs^vi^—Sr—N2^iii^	36.60 (5)
Cs^xii^—Cs—C3^iii^	43.02 (8)	Cs^vi^—Sr—N3^i^	109.90 (5)
Cs^xii^—Cs—N3^iii^	42.36 (10)	Cs^vi^—Sr—N3^vi^	70.10 (5)
Sr—Cs—Sr^ii^	108.15 (15)	N1—Sr—N1^iv^	180
Sr—Cs—Sr^i^	113.18 (5)	N1—Sr—N2^v^	90.4970 (12)
Sr—Cs—Sr^viii^	104.58 (4)	N1—Sr—N2^iii^	89.5030 (12)
Sr—Cs—C1	47.71 (5)	N1—Sr—N3^i^	90.1559 (18)
Sr—Cs—C1^i^	80.76 (9)	N1—Sr—N3^vi^	89.8441 (18)
Sr—Cs—N1	29.36 (6)	N1^iv^—Sr—N2^v^	89.5030 (12)
Sr—Cs—N1^i^	88.41 (8)	N1^iv^—Sr—N2^iii^	90.4970 (12)
Sr—Cs—C2^ii^	149.38 (7)	N1^iv^—Sr—N3^i^	89.8441 (18)
Sr—Cs—N2^ii^	130.69 (6)	N1^iv^—Sr—N3^vi^	90.1559 (18)
Sr—Cs—C3^iii^	78.33 (11)	N2^v^—Sr—N2^iii^	180
Sr—Cs—N3^iii^	88.72 (14)	N2^v^—Sr—N3^i^	89.952 (2)
Sr^ii^—Cs—Sr^i^	114.82 (5)	N2^v^—Sr—N3^vi^	90.048 (2)
Sr^ii^—Cs—Sr^viii^	105.80 (4)	N2^iii^—Sr—N3^i^	90.048 (2)
Sr^ii^—Cs—C1	154.37 (18)	N2^iii^—Sr—N3^vi^	89.952 (2)
Sr^ii^—Cs—C1^i^	91.92 (10)	N3^i^—Sr—N3^vi^	180
Sr^ii^—Cs—N1	136.3 (2)	C1—Fe—C1^vii^	180
Sr^ii^—Cs—N1^i^	105.47 (9)	C1—Fe—C2	90.5021 (17)
Sr^ii^—Cs—C2^ii^	84.45 (6)	C1—Fe—C2^vii^	89.4979 (17)
Sr^ii^—Cs—N2^ii^	94.42 (6)	C1—Fe—C3	90.4341 (12)
Sr^ii^—Cs—C3^iii^	48.54 (6)	C1—Fe—C3^vii^	89.5659 (12)
Sr^ii^—Cs—N3^iii^	31.08 (4)	C1^vii^—Fe—C2	89.4979 (17)
Sr^i^—Cs—Sr^viii^	109.57 (15)	C1^vii^—Fe—C2^vii^	90.5021 (17)
Sr^i^—Cs—C1	86.72 (10)	C1^vii^—Fe—C3	89.5660 (12)
Sr^i^—Cs—C1^i^	50.37 (4)	C1^vii^—Fe—C3^vii^	90.4341 (12)
Sr^i^—Cs—N1	99.12 (9)	C2—Fe—C2^vii^	180
Sr^i^—Cs—N1^i^	33.14 (5)	C2—Fe—C3	90.387 (2)
Sr^i^—Cs—C2^ii^	84.36 (13)	C2—Fe—C3^vii^	89.613 (2)
Sr^i^—Cs—N2^ii^	94.50 (16)	C2^vii^—Fe—C3	89.613 (2)
Sr^i^—Cs—C3^iii^	163.24 (9)	C2^vii^—Fe—C3^vii^	90.387 (2)
Sr^i^—Cs—N3^iii^	145.89 (8)	C3—Fe—C3^vii^	180
Sr^viii^—Cs—C1	77.56 (8)	Cs—C1—Cs^viii^	99.91 (7)
Sr^viii^—Cs—C1^i^	158.40 (18)	Cs—C1—Fe	112.69 (12)
Sr^viii^—Cs—N1	86.35 (7)	Cs—C1—N1	68.07 (12)
Sr^viii^—Cs—N1^i^	140.12 (19)	Cs^viii^—C1—Fe	103.06 (12)
Sr^viii^—Cs—C2^ii^	44.81 (5)	Cs^viii^—C1—N1	75.62 (12)
Sr^viii^—Cs—N2^ii^	26.11 (2)	Fe—C1—N1	178.6228 (6)
Sr^viii^—Cs—C3^iii^	77.49 (3)	Cs—N1—Cs^viii^	107.31 (6)
Sr^viii^—Cs—N3^iii^	88.16 (4)	Cs—N1—Sr	109.13 (13)
C1—Cs—C1^i^	91.87 (6)	Cs—N1—C1	93.41 (13)
C1—Cs—N1	18.52 (2)	Cs^viii^—N1—Sr	95.82 (12)
C1—Cs—N1^i^	84.74 (7)	Cs^viii^—N1—C1	86.01 (12)
C1—Cs—C2^ii^	112.70 (17)	Sr—N1—C1	155.5560 (16)
C1—Cs—N2^ii^	97.66 (14)	Cs^xiii^—C2—Fe	110.22 (4)
C1—Cs—C3^iii^	109.87 (10)	Cs^xiii^—C2—N2	69.56 (4)
C1—Cs—N3^iii^	126.34 (13)	Fe—C2—N2	178.6331 (10)
C1^i^—Cs—N1	89.26 (7)	Cs^xiii^—N2—Sr^xv^	117.29 (5)
C1^i^—Cs—N1^i^	18.369 (10)	Cs^xiii^—N2—C2	91.71 (5)
C1^i^—Cs—C2^ii^	127.70 (13)	Sr^xv^—N2—C2	150.6891 (7)
C1^i^—Cs—N2^ii^	143.06 (18)	Cs^xvi^—C3—Fe	120.47 (5)
C1^i^—Cs—C3^iii^	124.0 (2)	Cs^xvi^—C3—N3	59.13 (5)
C1^i^—Cs—N3^iii^	113.08 (18)	Fe—C3—N3	179.4092 (8)
N1—Cs—N1^i^	87.85 (6)	Cs^xvi^—N3—Sr^viii^	106.31 (4)
N1—Cs—C2^ii^	127.37 (16)	Cs^xvi^—N3—C3	103.40 (4)
N1—Cs—N2^ii^	110.12 (12)	Sr^viii^—N3—C3	150.1635 (8)
N1—Cs—C3^iii^	96.46 (12)		

Symmetry codes: (i) −*x*+1/2, *y*−1/2, −*z*+1/2; (ii) *x*+1, *y*, *z*; (iii) *x*+1/2, −*y*+1/2, *z*+1/2; (iv) −*x*, −*y*, −*z*+1; (v) −*x*−1/2, *y*−1/2, −*z*+1/2; (vi) *x*−1/2, −*y*+1/2, *z*+1/2; (vii) −*x*, −*y*, −*z*; (viii) −*x*+1/2, *y*+1/2, −*z*+1/2; (ix) −*x*+3/2, *y*−1/2, −*z*+1/2; (x) −*x*+3/2, *y*+1/2, −*z*+1/2; (xi) −*x*+1, −*y*, −*z*; (xii) −*x*+1, −*y*, −*z*+1; (xiii) *x*−1, *y*, *z*; (xiv) *x*−1/2, −*y*−1/2, *z*+1/2; (xv) −*x*−1/2, *y*+1/2, −*z*+1/2; (xvi) *x*−1/2, −*y*+1/2, *z*−1/2.
