# Supplementary figures and images for: Crystal structure of dicaesium strontium hexa­cyanidoferrate(II), Cs2Sr[Fe(CN)6], from laboratory X-ray powder data
Source: Acta Crystallogr E Crystallogr Commun. 2020 May 22;76(Pt 6):900–4. doi: 10.1107/S2056989020006660 (PMC7274004; doi:10.1107/S2056989020006660)

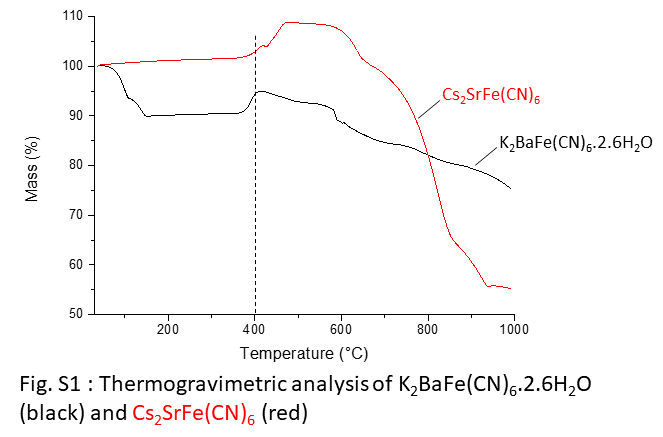

Supplement: Supplementary file 2 [file e-76-00900-sup2.png]
